# Associations of health literacy, personality traits, and pro-individualism with the willingness to complete advance directives in Taiwan

**DOI:** 10.1186/s12904-023-01215-3

**Published:** 2023-07-10

**Authors:** Duan-Rung Chen, Hui-Ching Weng

**Affiliations:** 1 Institute of Health Behaviors and Community Sciences, College of Public Health, National Taiwan University, Taipei, Taiwan; 2 Population Health Research Center, College of Public Health, National Taiwan University, Taipei, Taiwan; 3 Institute of Allied Health Sciences, College of Medicine, National Cheng Kung University, Tainan, Taiwan; 4 Institute of Gerontology, College of Medicine, National Cheng Kung University, Tainan, Taiwan

**Keywords:** Advance directive, Health literacy, Noncognitive factors

## Abstract

**Background:**

Studies indicated that patients with advance directives (ADs) have a generally better quality of life near death. Yet, the concept of ADs is relatively new in East Asian countries. This study examined the associations between health literacy, pro-individualism in end-of-life (EOL) decisions (i.e., EOL pro-individualism), and master-persistence personality traits with the willingness to complete ADs.

**Methods:**

The data is from a representative data of 1478 respondents from the 2022 Taiwan Social Change Survey. Generalized structural equation modeling (GSEM) was used to conduct path analysis.

**Results:**

Nearly half of the respondents (48.7%) were willing to complete ADs. Health literacy has direct and indirect effects through EOL pro-individualism values on the willingness to complete ADs. Noncognitive factors such as mastery-persistence personality traits and EOL pro-individualism values enhanced the willingness to complete ADs.

**Conclusion:**

A personalized communication strategy, mindful of personality dimensions and cultural values, can address individual fears and concerns, promoting the benefits of advance care planning (ACP). These influences can provide a roadmap for healthcare providers to customize their approach to ACP discussions, improving patient engagement in AD completion.

## Introduction

End-of-life (EOL) decisions are commonly arranged through advance directives (ADs), legal documents that outline treatment preferences, or by designating power of attorney to ensure patients receive care consistent with their wishes when incapacitated. Patients in the United States using ADs are more likely to receive care consistent with their preferences and tend to receive less aggressive EOL care [1]. They have a generally better quality of life near death [2].

Although patient autonomy has long been subject to legal protection in Western countries, such as that provided by the Patient Self-Determination Act passed by the US Congress in 1990, the Mental Capacity Act passed in the United Kingdom in 2005 [3, 4], the rate of advance care planning remains relatively low in the United States [5]. A systematic review examining research between 2011 and 2016 indicated that only 36.7% of US adults have ADs [6]. In the United Kingdom, the estimated rate of adults with ADs is about 4% in England and just 2% in Wales [7], whereas, in Germany, about 10% of the general population has ADs [8]. As of 2016, Medicare has agreed to pay physicians to engage in EOL conversations with their patients to help them to complete ADs [9].

The concept of ADs is relatively new in East Asian countries, among which Taiwan was the first to implement the Patient Autonomy Act (PAA) in 2019 to protect a patient’s right to natural death; it allows individuals aged ≥ 20 years to make decisions through advance care planning and complete a written AD to decline medical treatments in specific clinical scenarios in cases of incapacity [10]. Until the end of 2021, less than 0.2% of the adult population (35,545 of approximately 19.39 million adults) had completed an AD in Taiwan [11]. Low completion rates of ADs despite favorable attitudes toward ADs suggest that individual differences beyond access to information may influence the decision not to complete an AD [12].

An AD includes instruments such as living wills or durable power of attorney for health care. However, there is a growing awareness that ADs are limited because they do not capture the full range and scope of the multiple behaviors that make up the advance care planning process [13]. ACP has been redefined as an ongoing process consisting of several discrete behaviors, including contemplating, preparing, discussing, and readiness to complete the documents instead of a one-time advance directive document [14, 15]. This shift in perspective encourages continuous reflection on factors associated with the different stages of advance care planning behaviors, including the intention to engage in AD completion.

Therefore, envisioning ADs as a process emphasizes the significance of individuals’ continuously reflecting upon and expressing their values, beliefs, and preferences regarding end-of-life care. The cultural, religious, or personal value system heavily influences people’s perspectives on end-of-life decisions and how much family and healthcare providers collaborate on future healthcare decisions. Understanding these individual differences is essential when discussing and implementing ADs.

### The purpose of this study

This study examined (1) the associations of health literacy, mastery-persistence personality traits, and EOL pro-individualism with the willingness to complete ADs and (2) whether EOL pro-individualism mediates the association between health literacy and the willingness to complete ADs, as well as the associations between mastery-persistence personality traits and the willingness to complete ADs.

In this study, the phrase “EOL pro-individualism” emphasizes self-determination, autonomy, and preferences, specifically in the context of end-of-life decisions.

### Factors associated with willingness to complete ADs

#### Sociocultural norms

ADs allow competent individuals to exercise patient autonomy over medical care in anticipation of future incapacity [10]. However, the concept of ADs adopts a Western individualist framework that assumes people are autonomous and self-responsible individuals with unique views of proper conduct that must be respected [16]. Individualist values include autonomy, freedom, self-fulfillment, assertiveness, and personal uniqueness [16]. The values underlying ADs may differ from family-centered cultures deeply rooted in countries with Confucian traditions, such as Taiwan. Confucianism values familial responsibility and connection, and medical decisions regarding EOL decisions are highly family-centered [17, 18]. Familism (or collectivism) is considered more typical of non-Western societies and focuses on interpersonal relationships that promote group harmony through appropriate roles, duties, and obligations [19–21]. Given the divergence of culture between East Asia and the West, sociocultural value is critical in determining the acceptance of advanced care planning or ADs in East Asia [18, 21, 22]. An individual’s adapting to sociocultural norms may influence attitudes regarding advance care planning and the willingness to complete an AD [18]. Therefore, we hypothesize that people who express EOL pro-individualism values, as opposed to pro-familism/collectivism values, would be more willing to complete ADs.

#### Health literacy

Several studies have reported that knowledge of hospice and palliative care is the main factor associated with the completion of ADs [23–26]. However, few studies have examined the role of functional health literacy in the general population’s willingness to participate in advance care planning [25, 27]. Health literacy entails people’s knowledge, motivation, and competencies to access, understand, appraise, and apply health information to make judgments and medical decisions [28]. A systematic review reported that low health literacy predicts many disadvantaged outcomes, such as diabetes, sexually transmitted diseases, dental issues, and cardiovascular disease [29]. Studies also noted that people with poor health literacy were more passive in seeking health information [30] and less adept at communicating with medical staff [30]. They seldom express their ideas in shared decision-making processes [31], prefer to let doctors make decisions, and have lower participation in decision-making [32].

Health literacy is critical in understanding, completing, and using advance directives. Low health literacy is associated with insufficient advanced care planning knowledge [12]. It can significantly impact an individual’s ability to make informed decisions regarding EOL care [27]. This includes understanding complex medical terms, the implications of life-sustaining treatments, and effectively communicating preferences to healthcare providers [27]. Hence, we hypothesized that health literacy would be associated with the willingness to complete ADs.

Additionally, health literacy is necessary to grasp ADs’ ethical and legal aspects, and understanding those aspects may affect individuals’ value orientation toward EOL decisions. As indicated above, people with limited health literacy seldom express their values in shared decision-making processes [31] and have lower participation [32]. Hence, we hypothesized that people with high health literacy might be more likely to express self-determination value related to EOL decisions and, consequently, the willingness to complete ADs.

#### Personality traits

A systematic review emphasized the role of noncognitive factors in academic, psychosocial, cognitive, and health outcomes [32]. Specific personality and psychological traits have been identified as noncognitive factors. For example, grit, tenacity, curiosity, attitudes, self-control, self-efficacy, emotion, motivation, values, perseverance, delay of gratification, and confidence are noncognitive factors related to academic performance [32]. Noncognitive factors are reported to be equally or even more important than cognitive factors in the educational process [32]. Previous research has identified several noncognitive factors are predictors of advance care planning. For example, some studies have reported that life satisfaction, measuring a person’s well-being, relationship satisfaction, and self-perceived ability to cope with life, is related to willingness to complete ADs [32, 33]. Another study reported that dignity and personal control, rather than familial influence, were the primary and most influential motivating factors for completing ADs [34].

However, the association between noncognitive factors such as personality traits and ACP has yet to be well-established [35]. Personality traits are enduring characteristics that determine an individual’s pattern of thought, emotion, and behavior [36]. The most widely accepted model of personality, the Big Five, encompasses five core traits: openness, conscientiousness, extraversion, agreeableness, and neuroticism [37]. One recent study indicated that people engaged in advance care planning scored high on extroversion and conscientiousness scales [35]. Extraversion’s sociability and assertiveness characteristics can positively and negatively influence ACP. On the one hand, extroverted individuals’ strong communication skills can benefit ACP discussions. On the other hand, a preference for present-oriented enjoyment may lead such individuals to avoid contemplating negative future scenarios [38].

People with conscientiousness personality traits were more likely to engage in ACP [35]. People with high conscientiousness tend to plan, consider the future, and think through the potential consequences of their actions [37]. Conscientiousness represents the traits of being careful or diligent. It encompasses qualities like self-discipline, orderliness, and a strong sense of duty, implying a desire to achieve striving and to take obligations to others seriously [39]. Nevertheless, as Moon indicated, people with consciousness personality may differentiate themselves in their focus, whether dutifulness leans towards others, namely others-centered, whereas achievement striving is fundamentally self-centered [39].

The relationship between personality traits and cultural values is complex [40]. Cultural norms and values can shape the display and interpretation of personality traits [41]. Among people favoring collectivist cultures, traits like persistence, agreeableness, and conscientiousness may be more pronounced toward harmonious interpersonal relationships and responsibility toward the community. However, in people appreciating individualist cultures, high conscientiousness is associated with the organization, self-disciple, and achievement-striving mindset [42].

In this study, we created a conceptual construct of “mastery-persistence personality traits”, similar to the consciousness personality trait. In psychology, “mastery” is a crucial concept related to motivation, learning, and development and often refers to an individual’s orientation, goal, or motivation to gain competence and control over situations [43]. This is also an essential aspect of self-efficacy. “Persistence” is a personality trait that describes the ability to continue to work towards a goal or target despite facing challenges, difficulties, or discouragements. It is often associated with resilience, determination, tenacity, and endurance. As a result, “mastery-persistence personality” traits could potentially refer to an individual who has a strong desire to improve and grow, combined with a tenacity to stick with their goals even when faced with challenges. They likely have a mindset similar to consciousness and demonstrate resilience and perseverance. Therefore, we hypothesized mastery-persistence personality traits would be associated with willingness to complete ADs. In addition, given that people in Asia culture tend to be family-centered, people with a mindset similar to conscientiousness are likely to make decisions about their future health care beforehand to avoid burdening their family [12], which may be conducive to ACP engagement [44].

The relationship between mastery-persistence personality traits and individualist values may be intriguing. For instance, a person is inclined to persist in their endeavors, especially when those endeavors align with their personal beliefs, goals, or values; they are more likely to persist in achieving those goals, even in the face of difficulty or opposition. They may strongly value individualism. On other other hand, individualist values can create challenges to persistence when faced with a task that requires collective effort or conformity to cultural norms, such as when dealing with issues related to EOL decisions. Therefore, we hypothesized that mastery-persistence personality traits might be negatively related to EOL pro-individualism in a society toward collectivist culture such as Taiwan and may consequently affect the willingness to complete ADs.

### Socio-demographic factors and willingness to complete ADs

Willingness to sign an AD is positively associated with women [45, 46], older age [47], higher educational attainment [48], white ethnicity [49], and comprehension of the illness condition [50, 51]. For example, women reportedly anticipated the benefits of ADs and believed that having one could prevent the unwanted use of mechanical life support [45]. Therefore, we controlled for demographic variables, including sex, age, marital status, education, employment, whether they had children under 18 years, family members currently in long-term care (mentally ill, disabled, or frail), and illness histories such as cancer and mental illness in the path models. We also controlled whether people were aware of legislation regarding Patient Autonomy Act since 2019 (*yes* or *no*).

### The hypotheses

**Fig. 1:**
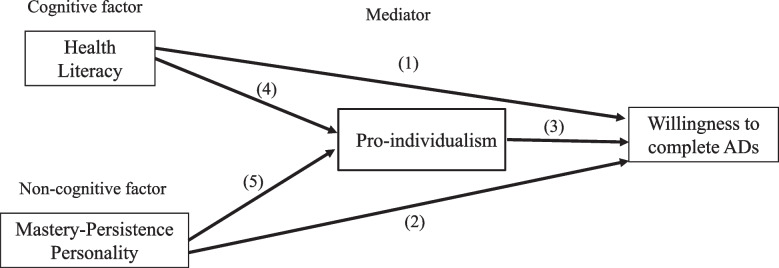
The pathways to the willingness to complete advance directives

We hypothesized that a) health literacy increases the willingness to complete ADs (path 1); b) mastery-persistence personality increases the willingness to complete ADs (path 2; c) EOL pro-individualism increases the willingness to complete ADs (path 3); d) health literacy increases EOL pro-individualism (path 4); e) mastery-persistence personality decreases EOL pro-individualism (path 5) (see Fig. 1).

## Methods

### Study design and sample

This study uses public data from the Taiwan Social Change Survey (Health Module) collected between September 2021 to April 2022. These data consist of a nationally representative sample. Samples were selected using multistage stratified multi-stage probability proportional to size sampling. The methodology of the TSCS has been described elsewhere [52]. Face-to-face interviews were conducted to collect the data using a structured questionnaire. Interviewers were required to attend a standardized 2-day training workshop before conducting interviews. Details of Taiwan Social Change Survey data are available on the Survey Research Data Archive website (https://srda.sinica.edu.tw/browsingbydatatype_result.php?category=surveymethod&type=1&csid=2; https://www2.ios.sinica.edu.tw/sc/en/home2.php).

The data were derived from individuals aged ≥ 18 years in Taiwan. There were 1604 respondents surveyed. The valid response rate was 43%. However, fifty-one respondents didn’t have income information, and variables of interest have missing values, including the willingness to complete ADs (*n* = 45), health literacy (*n* = 10), mastery-persistence personality (*n* = 30), pro-individualism (*n* = 26), and whether being aware of the PAA (*n* = 45). A total of 126 respondents were excluded. As a result, 1478 (1478/1604 = 92.1%) respondents were included in this study.

### Analysis plan

First, univariate analyses were conducted to examine the distributions of variables of interest. Second, exploratory factor analysis and reliability tests were conducted to explore the variables measured through multi-item constructs, including health literacy and mastery-persistence personality.

Because the dependent and mediating variables were categorical, we conducted a path analysis using generalized structural equation modeling (GSEM) to test our hypotheses. The goodness-of-fit index is not reported in generalized structural equation models. We reported 2000 bootstrapping adjusted odds ratios (aOR) and a 95% confidence interval. All statistical analyses were performed using Stata 16 (StataCorp, College Station, TX, USA).

All methods were carried out by relevant guidelines and regulations.

### Measures

#### Outcome variable

##### Willingness to complete ADs

Respondents were asked whether they would complete a written AD form to indicate future medical treatments in certain clinical conditions, such as terminal-stage diseases or incapacitation. Answers were scored on a 5-point Likert scale, and the responses were rated as 1 (*no*), 2 (*probably not*), 3 (*unsure*), 4 (*probably yes*), or 5 (*yes*). Respondents who answered *yes* were coded as being willing to complete ADs.

#### Explanatory variables

*Health literacy* was measured using the Brief Health Literacy Screen (BRIEF) [53, 54], a validated tool used in clinical settings. The BRIEF consists of four questions: (1) “How confident are you in filling out medical forms by yourself?” (2) “How often do you have problems learning about your medical condition because of difficulty understanding written information?” (3) “How often do you have problems understanding what is told to you about your medical condition?” (4) “How often do you have problems learning about your medical condition because of difficulty asking questions?” Answers are scored on a 5-point Likert scale, and the responses are rated as 1 (*always*), 2 (*often*), 3 (*sometimes*), 4 (*occasionally*), or 5 (*never*). Responses are reversed and summed into a possible score of 4 to 20 points, with higher numbers representing higher health literacy levels [53, 55]. Health literacy is a continuous variable. Details of the scale are presented in Table 1.

**Table 1:** Multi-item cognitive-affective constructs (*N* = 1478)

Construct	Factor loading	Cronbach’s α	Variance explained
**Health literacy**		0.793	63.22%
How often do you have problems learning about your medical condition because of difficulty understanding written information without help?	0.824		
How often do you have problems learning about your medical condition because of difficulty with what is told to you by health professionals without help?	0.859		
How often do you have problems learning about your medical condition because of difficulty asking questions without help?	0.843		
How confident are you in filling out medical forms by yourself without help?	0.633		
**Mastery-persistence personality**		0.71	63.64%
Even if I feel sick enough to justify taking sick leave, I still work hard to complete the work I need daily.	0.799		
Even though I dislike the work, I still do my best.	0.805		
Even if it takes a long to see results, I continue to do my best.	0.789		

*Mastery-persistence personality* was measured using three questions asking the respondents if they agreed with statements describing who they are. These three items each represented a different aspect of noncognitive skills [32]: (1) persistence is defined as being able to maintain a course of action or persevere with a task and finish it despite the obstacles (such as opposition or discouragement) or the effort involved. It was measured by asking respondents whether they agree with the statement: “Even if I feel sick enough to justify taking sick leave, I still work hard to complete the work I need to do every day.” 2) Self-control was defined as the ability to command one’s behavior (overt, covert, emotional, or physical) and to restrain or inhibit one’s impulses. It was measured by asking respondents to respond to the statement, “Even if I dislike the work, I still do my best.” 3) Delay of gratification was defined as the ability to forgo immediate reward for a greater future reward. It was measured using the statement, “Even if it takes a long time to see results, I continue to do my best.”

Answers to these above three questions were scored on a 4-point Likert scale, and the responses are rated as 1 (*strongly disagree*), 2 (*disagree*), 3 (*agree)*, or 4 (*strongly agree*). Exploratory factor analysis was performed, and one factor with an explained variance of 63.64% was observed and labeled as a “mastery-persistence personality”. This factor had good internal reliability with a Cronbach’s α value 0.713. We summed the ratings of these three items, which yielded scores ranging from 3 to 12 [55]. A higher score indicated a stronger tendency to have a mastery-persistence personality. Details are presented in Table 1.

*EOL pro-individualism* was measured through responses to a scenario describing having to make decisions in EOL situations. Respondents were asked, “Suppose you were diagnosed with terminal cancer; how would you decide to inform you of the diagnosis and treatment plans?” Respondents can choose one of five answers: (1) *Only inform my next of kin, and the person can ultimately decide for me*; (2) *Inform my next of kin and me, and the person will consult with me and make the decision for me*; (3) *Inform my next of kin and me. The person will consult with me, and we will make the decision together*; (4) *Inform my next of kin and me. I will consult with the person, but I will make the decision myself*; (5) *Only inform me, and I can make my own decision*. People who chose answers 4 or 5 were coded “1” as “EOL pro-individualism,” and the remaining choices were coded “0” as “EOL pro-familism/collectivism”.

#### Control variables

*Sex* was coded as 1 for women and 0 for men.

*Education level* was defined as the highest diploma respondents obtained. Education level was categorized as junior high school and below (compulsory education), high school graduates or equivalents, and college graduates and graduate school graduates. Junior high school and below were considered as the reference group.

*Personal monthly income level* This survey includes 23 monthly income categories, ranging from no income to over 300,000 NTD (approximately 10,000 USD), which was treated as a continuous variable.

*Age* was divided into four groups. Young adults aged 18 to 34 years old, adults aged 35 to 44 years old, middle-aged adults aged 45 to 64, and older adults aged above 65 years old. Young adults were considered as the reference group.

*Marital status* was coded as single or never married, divorced or separated, or widows. A small number of respondents (*n* = 37) reported they were currently living with others. We recorded their marital status according to their previous marital status. As a result, 23 respondents were recorded as single or never married, 12 as divorced, and 2 as widows. Single or never married respondents were treated as the reference group.

*Employment status* was coded as full-time employment, part-time or working for a family business, unemployed, and respondents who were currently retired, students, or serving the army were grouped and formed the reference group.

*Family members currently in long-term care* were recorded by asking respondents whether their family members were currently in long-term care because of chronic diseases, mental illness, or frailty with an answer format of *yes* or *no*.

*Awareness of the legislation of PAA* Respondents was asked if they knew the PAA had already been legislated and implemented. The answer format was *yes* or *no*.

*Respondents were asked whether they had cancer,* and the answer format was yes or no.

*Whether had mental illness* Respondents were asked if they had a mental illness, and the answer format was yes or no.

## Results

A total of 1478 respondents were in the analysis. Table 2 presents the demographic characteristics of the respondents. Of the study respondents, 52.9% were women, 38.6% had a college education or higher, and the mean age was 47.9. Half the respondents were married (54.2%), and approximately 29.1% were single. The mean personal monthly income ranged from USD 978 to USD 1304. Over half of the respondents had full-time jobs (59.1%), and about 27.6% were retired or still in school or the army. Approximately one-third (31%) had children under 18 years. Nearly half (48.7%) of respondents were willing to complete ADs. A small percentage of the respondents revealed they had cancer or mental illness (1.6% and 1.1%, respectively; more details are provided in Table 2).

**Table 2:** Characteristics of the respondents (*N* = 1478)

Variables	N (Mean)	%/SD
Women	772	52.2
Education level		
Junior high school and below	329	22.3
High school and equivalents	579	39.2
College	445	30.1
Graduate school	125	8.5
Marital status		
Single	429	29.1
Married	802	54.2
Separate/divorced	149	10.1
Windowed	98	6.6
Employment		
Full-time	873	59.1
Part-time or family business	71	4.8
Retired, students or military	409	27.6
Unemployed	125	8.5
Personal monthly income (in US dollars)^a^	USD 978–1304	USD 326–652
Age group	47.9	15.9
Young adults (18–34)	350	23.7
Adults (35–44)	294	19.9
Middle-aged adults (45–64)	575	38.9
Older adults (65+)	259	17.5
Had chidern under 18yrs (Yes)	458	31.0
Had cancer (Yes)	23	1.6
Had mental problem (Yes)	16	1.1
Have family members currently in long-term care	901	61.0
Health literacy (4–20)	17.44	12.6
Pro-individualism (Yes)	622	42.1
Mastery-persistence personality (3–12)	9.08	1.3
Aware of the Patient Autonomy Act (Yes)	548	37.1
Willing to complete ADs (Yes)	720	48.7

Rounding differences to 100% are possible

^a^During the month of June 2023, the New Taiwan Dollar to US Dollar exchange rate is recorded at 30.67

Table 3 presents the path analysis results using GSEM. Health literacy was positively related to the likelihood of willingness to complete ADs (aOR = 1.148, *p* < 0.0001) (path 1), and mastery-persistence personality was also significantly associated with the likelihood of willingness to complete ADs (aOR = 1.113, *p* < 0.02) (path 2). In addition, EOL pro-individualism increases the chance of willingness to complete ADs (aOR = 1.354, *p* < 0.01) (path 3). The result suggests that with one unit increase in health literacy score, the chance of willingness to complete ADs increases by 14.8%, and with one unit increase in mastery-persistence personality score, the chance of willingness to complete ADs increases by 11.3%. In addition, compared to people who did not express EOL pro-individualism value, people who share this value are 35.4% more likely to complete ADs.

**Table 3:** Generalized path analysis results for willingness to complete ADs (*N* = 1478)

**Expouse variables**	**Pro-individualism (Mediator)**				**AD (Outcome variable)**			
**Pathways**	Coeff. (log odds)	aOR	95% CI	*P*- value	Coeff. (log odds)	aOR	95% CI	*P*- value
Health literacy → Willingness to complete ADs (path 1)					0.138	1.148	(1.093, 1.206)	0.00
Mastery-Persistence personality → Willingness to complete ADs (path 2)					0.107	1.113	(1.016, 1.220)	0.02
Pro-individualism → Willingness to complete ADs (path 3)					0.303	1.354	(1.072, 1.711)	0.01
Health literacy → Pro-individualism (path 4)	0.064	1.066	(1.022, 1.112)	0.003				
Mastery-persistence personality → Pro-individualism (path 5)	-0.069	0.934	(0.861, 1.012)	0.095				
**Control variables**								
Women (ref: men)					0.232	1.261	(0.985, 1.614)	0.11
Marital status (ref: singles)								
Married					0.438	1.550	(1.101, 2.183)	0.01
Separated/ Divorced					0.383	1.466	(0.959, 2.241)	0.07
Widowed					0.551	1.735	(0.956, 3.149)	0.07
Education level (ref: Junior high school & below)								
High schools or equlivents					0.037	1.038	(0.753, 1.431)	0.82
College					0.034	1.034	(0.703, 1.522)	0.86
Graduate Sch					-0.334	0.716	(0.419, 1.223)	0.21
Employment (ref: Retired, students or others)								
Full-time					-0.257	0.773	(0.552, 1.082)	0.13
Part-time or family business					-0.487	0.614	(0.369, 1.022)	0.06
Unemployed					-0.235	0.790	(0.436, 1.434)	0.42
Income					0.009	1.009	(0.973, 1.046)	0.59
Age (ref:Young adults 18–34)								
Adults (35–44)					0.746	2.107	(1.464, 3.035)	0.00
Middle-aged adults (45–64)					0.976	2.654	(1.833, 3.842)	0.00
Older adults (65+)					0.709	2.032	(1.201, 3.438)	0.01
Have childern under 18yrs (ref: No)								
Yes					-0.406	0.667	(0.506, 0.878)	0.00
Family members in long-term care (ref: No)								
Yes					-0.192	0.826	(0.645, 1.057)	0.12
Awareness of the PAA legistiatoin (ref: No)								
Yes					0.540	1.715	(1.352, 2.177)	0.00
Have cancer (ref: No)								
Yes					1.440	4.221	(0.006, 2869.516)	0.04
Have mental illness (ref: No)								
Yes					1.155	3.173	(0.429, 23.501)	0.02

### Testing for the mediation effect of EOL pro-individualism

**Fig. 2:**
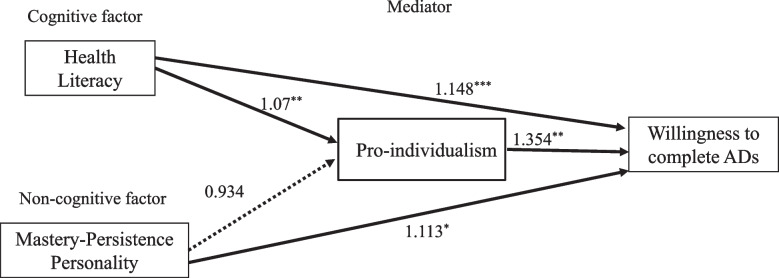
Generalized path analysis results (aORs are reported). The model has controlled for age, sex, education level, employment, personal income, children under 18, whether family members were in long-term care, had cancer, or mental illness, and were aware of the PAA

The results indicate that health literacy affected the likelihood of willingness to complete ADs through its positive association with EOL pro-individualism values (aOR = 1.066, *p* < 0.003) (path 4). However, mastery-persistence personality traits were not significantly associated with pro-individualism values (aOR = 0.934, *p* = 0.095) (path 5). Thus, EOL pro-individualism values partially mediate the association between health literacy and willingness to complete ADs. The result suggested that health literacy increases the willingness to complete ADs and, indirectly, through increasing EOL pro-individualism, enhances the willingness to complete ADs. The master-persistence personality was negatively associated with EOL pro-individualism, yet it didn’t reach a statistically significant level (*p* = 0.095). Figure 2 illustrates the generalized path analysis results.

### Control variables associated with willingness to complete AD

Compared with young adults (aged 18–34), respondents of older-aged cohorts were all more likely to be willing to complete ADs. Middle-aged adults (45–64 years old) had the highest likelihood of willingness to complete ADs (aOR = 2.654, *p* < 0.001). Compared with single or never married respondents, married people were 55% more willing to consider the completion of ADs (aOR = 1.55, *p* = 0.01). In addition, people living with children under 18 were about 17.4% less likely to be willing to complete ADs (aOR = 0.667, *p* < 0.0001). Lastly, awareness of PAA legislation was 71.5% more likely to be willing to complete ADs (aOR = 1.715, *p* < 0.001). People with cancer or mental illness were significantly inclined to complete ADs (aOR = 4.221, *p* < 0.04; aOR = 3.173, *p* = 0.02, respectively).

## Discussion

This study examined the associations of health literacy, mastery-persistence personality traits, and EOL pro-individualism with the willingness to complete ADs. The results confirmed that health literacy increases the willingness to complete ADs (path 1), mastery-persistence personality traits increase the willingness to complete ADs (path 2), and c) EOL pro-individualism increases the willingness to complete ADs (path 3). The results further proved the mediating role of EOL pro-individualism in the associations of health literacy and the willingness to complete ADs, indicating health literacy has a positive direct effect on the willingness to complete ADs and a positive indirect effect through EOL pro-individualism (path 4) to enhance the willingness to complete ADs. Several notable findings were revealed.

First, this study observed that health literacy significantly predicts the willingness to complete ADs. EOL pro-individualism value partially mediates the effect of health literacy on the willingness to complete ADs. Additionally, health literacy can affect the willingness to complete ADs through EOL pro-individualism. Health literacy is how individuals obtain, process, and understand the basic health information needed to make appropriate health decisions. By enhancing health literacy, it is suggested that patients can better understand their health situation, their rights, and the potential consequences of different decisions. This understanding may help them feel more empowered to participate actively in their own EOL decisions, promoting a greater sense of self-determination. It could also potentially create a balance between the traditionally familial-oriented decision-making process and individual autonomy, especially in critical end-of-life situations in societies like Taiwan that traditionally place a higher value on familial expectations and consensus during end-of-life (EOL) decisions [21, 56]. Increasing health literacy in such societies can help enhance the sense of patient autonomy to signify a shift towards acknowledging and empowering the individual’s right to make their own end-of-life decisions. It may be beneficial to have conversations with families and society about the importance of patient autonomy and how it can coexist with familial expectations and decisions.

Several studies have reported that knowledge of hospice and palliative care [23, 24, 50, 57] is associated with the completion of ADs. Few studies have examined the role of functional health literacy in the willingness to complete ADs [25, 58]. For example, one study revealed that health literacy is a stronger predictor of completion of ADs than prior advanced care planning experience and advanced care planning knowledge [25]. Another study reported that participants with low or marginal health literacy were more likely than participants with adequate health literacy to have preferences for aggressive EOL care [59].

Individuals with limited health literacy may be less likely to participate in advance care planning because of difficulty comprehending complex health information [59]. People with limited literacy are often unsure about treatment decisions [60] and more passive in seeking health information [32]. They rarely express their ideas in shared decision-making processes [31] and prefer to let doctors make decisions. Communication barriers with healthcare systems rather than educational attainment are the primary reason health literacy is related to EOL care choice [59]. Our findings support that health literacy, not educational attainment, is more critical for predicting people’s intentions to complete ADs. A recent review on the effectiveness of advance care planning interventions adapted for limited health literacy showed that most studies were conducted in White patients in outpatient clinics in the United States [61]. They found that intervention programs for enhancing health literacy can increase ACP knowledge, preference for comfort care, engagement, and care documentation. However, more studies that address the effects of limited health literacy on advance care planning in diverse populations and settings are needed [61].

Higher health literacy positively influences the willingness to complete ADs. This finding implies that efforts should focus on populations with lower health literacy, potentially reducing health disparities. Culturally sensitive approaches and trust-building communication strategies are also crucial in this process. Clinicians should use materials appropriate for their patients’ health literacy levels to address their advance care planning needs. This could help to improve awareness and understanding of the importance of advance directives.

Second, this study observed that respondents who value individualism in EOL decisions were more willing to complete ADs. Systematic reviews have summarized the studies on EOL communication, including ADs and ACP, in the context of East Asian culture [21, 22]. They reported that people in Asian countries, especially in China with the influence of Confucianism, tend to have negative attitudes toward communication on dying and prefer clinician-centered approaches to decision-making or the prioritization of family expectations over patient autonomy in prognosis disclosure [22]. However, a study on patients in Korea, China, and Japan reported that most participants preferred making EOL care decisions alone despite the influence of family [62]. Older adults being informed of a terminal illness diagnosis preferred having ADs for medical treatment [63]. The current study found that respondents favoring self-determination and patient autonomy in EOL situations tend to engage in the completion of ADs. These studies revealed the complex dynamics between cultural values and individual choices in the ACP process.

Third, our study observed that respondents with master-persistence personality traits were more likely to complete ADs than their counterparts were. Master-persistence personality trait typically denotes an individual who is diligent and determined and maintains a persistent effort toward their goals, even in the face of obstacles or setbacks. Such individuals are usually disciplined, self-motivated, and show resilience. The completion of ADs requires an individual to confront the uncomfortable prospect of their mortality, make complex decisions about potential future healthcare scenarios, and engage in planning and paperwork. These tasks can be challenging and emotionally demanding. As such, it is understandable that individuals with master-persistence personality traits may be more likely to complete ADs than others.

Only a few studies examining the relationship between personality traits and the completion of ADs reported that respondents who engaged in advance care planning scored high on extroversion and conscientiousness [35, 64]. Individuals with conscientious personality traits exhibit self-discipline, carefulness, and a tendency to think carefully before acting [65]. Our study also revealed that respondents with a master-persistence personality displaying self-control, persistence, and delayed gratification were more willing to complete ADs. This finding confirms that personality traits similar to conscientiousness are related to acceptance of advance care planning, including willingness to complete ADs. Noncognitive factors such as personality traits are crucial for understanding people’s inclination to plan for their future care. This result implies that personality traits can impact health-related behaviors and decision-making. Therefore, understanding these connections might be valuable in developing strategies to encourage more people to complete their ADs.

We also observed that demographic backgrounds were significantly associated with willingness to complete ADs. Marital status or having children under age 18 were significantly associated with willingness to complete ADs. These findings confirm that having dependent children reduces the intention to complete ADs [57]. Worthing of noting, married people were 55% more willing to consider completing ADs than single or never married. Yet they have the lowest percentage expressing pro-individualism in EOL decisions, compared to divorced or separated respondents (37.5% vs. 58.4%, *P* < 0.0001). Given that people in Asia culture tend to be family-centered, married people may be more likely to complete ADs beforehand to avoid burdening their families [11]. They tend to the family’s needs over the individual’s [22]. Married people may feel more responsibility towards their families, leading them to demonstrate less pro-individualism in EOL decisions. However, they may be more likely to complete ADs to prevent causing distress or burden to their families. They may independently complete ADs out of concern for their loved one’s well-being, even if they align more with shared decision-making. On the other hand, single, divorced, or separated respondents might not have a spouse to lean on for EOL decisions, making them more likely to assert their individualist preferences in EOL situations. Advanced care planning programs and policies should aim to be culturally sensitive and flexible enough to accommodate individual preferences and values.

Additionally, awareness of the legislation of the PAA was significantly associated with willingness to complete ADs, indicating that a lack of understanding of the need for ADs remains a significant reason why many individuals do not have one [66]. Lastly, respondents with a history of serious illnesses such as cancer or mental illness also had higher intentions of completing ADs. This finding confirms that having cancer and mental conditions for extended periods predicts the completion of advanced care planning [58].

In summary, the concept and process of ADs can be challenging for many individuals, particularly those with lower health literacy. By simplifying the language used in advance care planning materials, individuals with lower health literacy might find it easier to comprehend the information [67]. Second, some cultural norms and personal beliefs might make it more difficult for people to engage in discussions about death, illness, and end-of-life decisions [20, 22, 68]. This can be especially true for individuals with lower pro-individualism values who may prefer collective decision-making. For those with lower pro-individualism values, including family members, caregivers, or other significant individuals in the planning process might be helpful. This can help ensure decisions align with the person’s cultural or personal beliefs and values and helps make the planning process more of a shared responsibility [69, 70]. Third, people’s preferences for end-of-life care might change over time due to various factors such as changes in health status, personal experiences, or shifts in values and beliefs. For those with lower mastery-persistence personality, it is essential to present ACP as an iterative process rather than a one-time event. This means regular reviews and updates, providing continual support, and allowing decisions to change over time. As such, communication-based advance care planning interventions might indeed serve as a more accessible and inclusive approach. These interventions could employ more straightforward language, encourage continuous dialogue about care preferences, incorporate a broader range of cultural and individual perspectives, and promote regular updates in line with evolving personal circumstances and preferences [71, 72]. However, rigorous research is required to understand such interventions’ effectiveness and limitations fully. Future studies should consider these aspects and aim to enhance the inclusivity and accessibility of advance care planning. These research efforts could contribute to more inclusive and effective advance care planning practices.

### Limitations

This study had several limitations. First, the study employed a cross-sectional design, which precluded the determination of causal relationships. Second, the survey was conducted during the COVID-19 pandemic, resulting in a response rate below 50%. Third, the authors devised the measures of EOL pro-individualism and master-persistency personality traits employed in this study, which may have intrinsic limitations. While these measures were designed with careful consideration, the potential for biases, inaccuracies, or misinterpretations needs to be addressed. Fourth, the study examined respondents’ willingness to complete ADs; however, people’s preferences for EOL care choices are unstable over time [73]. A problem known as affective forecasting error [64] is that healthy people often cannot predict their preferences in the event of future illness. The instability of preferences over time, and the fact that an individual’s health status can shape these preferences, underlines the need for flexibility and ongoing communication in EOL care planning. Given that the preferences of healthy individuals can change substantially if they become seriously ill, EOL care plans must be adaptable to changing circumstances and the evolving wishes of the patient. In practice, this might mean encouraging patients to communicate their wishes with their loved ones and care providers regularly and educating patients about the possibility of preference changes so that they understand the importance of keeping the conversation open. Preference stability was generally stronger among inpatients and seriously ill outpatients than among adults without serious diseases [73]. Patients engaged in ACP had more preference stability, and preferences to forgo therapies were generally more stable than preferences to receive treatments [73]. Further research in real-world settings is required to confirm the utility of advanced care plans for future end-of-life decisions. Since we utilized public records for this study, Institutional Review Board (IRB) approval was not required. Lastly, the generalizability of the study’s findings to other countries may be limited due to using a sample derived exclusively from Taiwan; caution is necessary.

## Conclusion

Half of the respondents (*n* = 720, 48.7%) were willing to complete ADs. If combined with the respondents who were very likely to be ready to complete ADs (*n* = 461, 31.2%), most of the study respondents (*n* = 1181,79.9%) were willing to make EOL treatment decisions by completing an advanced directive. However, only approximately one-third of the respondents knew of the PAA [66], a new legitimization to protect patients’ right to natural death. Given that less than 0.2% of the adult population in Taiwan had completed an AD by the end of 2021, several factors could be contributing to the low completion rates, despite favorable attitudes toward them. In Taiwan, completing an AD requires discussing personal wishes with healthcare providers and family members, a mandatory ACP process, before completion of an AD. Access to these professionals may be limited for some people, and completing an AD may also be seen as complex or daunting. Additionally, some people might avoid making an AD because they have faith in their family to make the right decisions, or they may be uncomfortable deciding on their medical treatment in advance. To address these barriers, interventions like public education campaigns, simplifying the process of completing an AD, making legal support readily available, and encouraging open conversations about end-of-life care could be helpful. Healthcare professionals could be trained to initiate conversations about ADs with their patients to make the process more routine and less intimidating.

This study found that individual factors, including health literacy, mastery-persistence personality traits, and EOL pro-individualism values, are predictors of willingness to complete ADs. Tailoring the communication about the benefits of ACP to fit each patient’s unique situation could make the process more relevant and compelling. For example, for patients who value autonomy and control, the discussion could focus on how ACP allows them to maintain control over their healthcare decisions. By taking a personalized approach that considers each patient’s unique characteristics, healthcare providers can improve patient engagement in ACP, leading to higher AD completion rates and better end-of-life care outcomes. The current study can provide a roadmap for healthcare providers to customize their approach to ACP discussions, improving patient engagement in AD completion.

## Data Availability

The datasets used and/or analyzed during the current study are available from the corresponding author, Duan-Rung Chen, upon reasonable request (email: duan@ntu.edu.tw).

## Abbreviations

PAA: Patient Autonomy Act
ADs: Advance Directives
ACP: Advance Care Planning
EOL: End-of-Life
